# Orexins and bipolar disorder: A review

**DOI:** 10.1192/j.eurpsy.2022.1014

**Published:** 2022-09-01

**Authors:** C. Moya Lacasa, L. Gonzalez-Blanco, C. Martinez-Cao, C.M. Alvarez-Vazquez, E. Martin-Gil, A. García Fernández, P.A. Saiz, J. Bobes, M.P. Garcia-Portilla

**Affiliations:** 1 University of Oviedo, Department Of Psychiatry, Oviedo, Spain; 2 University of Oviedo, Department Of Psychiatry, oviedo, Spain

**Keywords:** hypocretin, circadian rhythms, bipolar disorder, orexins

## Abstract

**Introduction:**

Bipolar disorder (BD) is a chronic deteriorating illness which has a strong impact on functionality. In the past few years, orexins have gained importance as possible biomarkers of circadian rhythms, affected in BD. Up to this date, we have not found any bibliographical review evaluating the association of orexins and BD.

**Objectives:**

To review published literature in relation to the associaton of orexins and BD.

**Methods:**

A bibliographical search was conducted in PubMed. Inclusion criteria were a) the study evaluated orexins in plasma or cerebrospinal fluid, and b) patients with BD were included within the subjects of study.

Reference lists of the articles that met inclusion criteria were also examined.

**Results:**

Ten articles were retrieved from the initial search. Only three met inclusion criteria and another one was selected from the reference list examination. One study observed significantly higher levels of orexin A in plasma of BD patients versus depression and controls. Other found higher concentration of orexin A of unipolar and bipolar depression versus controls, but this result was not statistically significant. Another one did not find differences in orexin A concentration between mania, depression and controls. The remaining study detected significantly lower concentration of orexin A in BD versus depression, schizophrenia and controls.

**Conclusions:**

Despite being heterogenous, the results point out there are differences in orexin levels in BD when compared to other diagnostic groups or controls. This sets a starting point to focus research on this subject and continue analyzing the role of orexins as biomarkers in BD.

**Disclosure:**

No significant relationships.

